# Synaptic dysfunction and septin protein family members in neurodegenerative diseases

**DOI:** 10.1186/s13024-015-0013-z

**Published:** 2015-04-03

**Authors:** Mikael Marttinen, Kaisa MA Kurkinen, Hilkka Soininen, Annakaisa Haapasalo, Mikko Hiltunen

**Affiliations:** Institute of Biomedicine, University of Eastern Finland, P.O. Box 1627, 70211 Kuopio, Finland; Institute of Clinical Medicine – Neurology, University of Eastern Finland, Kuopio, Finland; Department of Neurology, Kuopio University Hospital, Kuopio, Finland

**Keywords:** Alzheimer’s disease, Frontotemporal lobar degeneration, Huntington’s disease long-term depression, Long-term potentiation, Neurodegeneration, Parkinson’s disease, Septin, Synaptic dysfunction, Synaptic plasticity

## Abstract

Cognitive decline and disease progression in different neurodegenerative diseases typically involves synaptic dysfunction preceding the neuronal loss. The synaptic dysfunction is suggested to be caused by imbalanced synaptic plasticity i.e. enhanced induction of long-term depression and concomitantly decreased long-term potentiation accompanied with excess stimulation of extrasynaptic *N*-Methyl-D-aspartate (NMDA) receptors due to various disturbances in pre- and postsynaptic sites. Recent research has identified neurodegenerative disease-related changes in protein accumulation and aggregation, gene expression, and protein functions, which may contribute to imbalanced synaptic function. Nevertheless, a comprehensive understanding of the mechanisms regulating synaptic plasticity in health and disease is still lacking and therefore characterization of new candidates involved in these mechanisms is needed. Septins, a highly conserved group of guanosine-5'-triphosphate (GTP)-binding proteins, show high neuronal expression and are implicated in the regulation of synaptic vesicle trafficking and neurotransmitter release. In this review, we first summarize the evidence how synaptic dysfunction is related to the pathogenesis of Alzheimer’s, Parkinson’s and Huntington’s disease and frontotemporal lobar degeneration. Then, we discuss different aspects of the potential involvement of the septin family members in the regulation of synaptic function in relation to the pathogenesis of neurodegenerative diseases.

## Introduction

Impaired function and degeneration of synapses are among the earliest pathological alterations in neurodegenerative diseases. The exact molecular mechanisms that cause synaptic dysfunction in neurodegenerative diseases remain unclear, but significant efforts have been invested on understanding disease-related alterations in synaptic structure, function, and plasticity. Synaptic plasticity is generally divided into two main forms, long-term potentiation (LTP) and long-term depression (LTD). LTP is a process linked to learning and memory formation. In LTP, coinciding activation of both pre- and postsynaptic elements take place, leading to a long-lasting increase in synaptic transmission between the terminals and persistent strengthening of the synapse [1]. LTD, in turn, is a process depressing synaptic activity for a prolonged time. It is believed that in several neurodegenerative disorders, LTP is disrupted and LTD is promoted [2-4]. The most prominent forms of LTP and LTD are *N*-Methyl-D-aspartate receptor (NMDAR)-dependent. Different patterns of synaptic activation result in NMDAR activation, leading to induction of either LTP or LTD, through regulation of α-amino-3-hydroxy-5-methyl-4-isoxazolepropionic acid receptor (AMPAR) localization at the postsynaptic terminal [5,6]. Overstimulation of extrasynaptic NMDAR is commonly noticed in neurological disorders, leading to an excess influx of calcium to the postsynaptic site, possibly promoting LTD and triggering various neurodegenerative events [7-9]. Also, observed variations in presynaptic and astrocyte activity have supported the idea for disease-related extrasynaptic NMDAR activation and LTP suppression [10,11]. Studying the relation between neurodegenerative disorders and alterations in synaptic plasticity is difficult due to that fact that the underlying mechanisms, which determine whether synaptic activation results in LTP or LTD, are not completely understood [6]. In addition, the neurodegenerative disease-associated mechanisms affecting the formation of LTP or LTD are thus far not clear. However, recent studies have identified several factors involved in neurodegenerative disorders, which may modulate synaptic plasticity, Although the understanding of synaptic function-related processes has recently leaped forward, unraveling the detailed mechanisms of synaptic function is essential for understanding the pathogenesis of neurodegenerative diseases at the molecular level. Recent studies have identified septin protein family members as possible candidates that take part in the regulation of synaptic processes and whose altered function might be involved in synaptic dysfunction in neurodegenerative diseases. Septins belong to a highly conserved family of guanosine-5'-triphosphate (GTP)-binding proteins, which play a role in the axonal transport, vesicular trafficking, and neurotransmitter release [10,12]. In addition, septins have been shown to interact with several key components related to neurological disorders (e.g. CDK5, XIAP/caspase-3, VAMP2, Parkin, and EAAT1). In this review, we will provide insights into synaptic dysfunction in neurodegenerative diseases, and how septins could play a role in the events leading to impaired synaptic function.

### Synaptic dysfunction in Alzheimer’s disease

Alzheimer’s disease (AD) is the most common neurodegenerative disorder in the world, which affects up to 50% of individuals above the age of 85. AD is clinically associated with a global cognitive decline and progressive loss of memory and reasoning. At autopsy, a large number of neuritic plaques and neurofibrillary tangles (NFT) in the neocortex of the brain are detected. These consist of amyloid-β (Aβ) peptide and hyperphosphorylated tau protein, respectively [13-16]. The Aβ peptide is released from APP after sequential proteolytic cleavage by β- and γ-secretases. The majority of APP is cleaved by α-secretases, which leads to the release of the neuroprotective ectodomain portion of APP (sAPPα) and prevents Aβ formation. Conversely, the cleavage of APP by β-secretase or BACE1 leads to the formation of the N-terminal secreted APPβ (sAPPβ) and an APP C-terminal fragment (CTF) C99, which is consequently cleaved by γ-secretase producing Aβ [17-20]. In AD, based on the prevailing amyloid cascade hypothesis, soluble Aβ peptide levels are drastically increased, augmenting synaptic dysfunction, calcium dyshomeostasis, inflammation, oxidative stress as well as tau hyperphosphorylation and the formation of NFTs at specific brain regions in AD [21-23]. Synapses are considered the earliest site of pathology, and reduced synaptic activity is found to be the best pathological correlate of cognitive impairment in Alzheimer's disease [24]. Therefore, it is proposed in the amyloid cascade hypothesis, that accumulation of Aβ is an initial trigger for AD. Recent *APP* mutation studies support the notion that increased Aβ production is a major factor causing AD. These studies identified a potential protective mutation in *APP* [25,26]. Substitution of alanine to threonine at position 673 in APP (A673T) was shown to decrease the production of Aβ by 50-fold [25]. Individuals with the mutation A673T on APP have decreased cognitive impairment due to aging and they score better in cognitive tests than those without the mutation. This raises the possibility that reduced Aβ production throughout an individual’s lifespan possibly has a protective effect against AD [25]. The exact molecular mechanisms of how Aβ accumulation initiates AD are unknown, and a focus has been set on unraveling the deleterious effects of excess Aβ on synaptic function. Recent studies have shed light on a variety of pathways, which Aβ synaptotoxicity is mediated through.

Despite the well-established foundation of the amyloid cascade hypothesis, the reported Aβ-targeted trials in AD patients to date have not been successful [27]. Therefore, alternative therapeutic approaches focusing on other key events, such as the hyperphosphorylation and aggregation of tau have been actively explored. Recent findings show that the soluble forms of tau are synaptotoxic [28], which is comparable to that observed with soluble Aβ oligomers [8,29,30]. Importantly, mislocalized tau in its hyperphosphorylated form has been shown to impair synaptic plasticity before the formation of NFTs [31,32]. However, failures in Aβ-targeted trials do not dismiss Aβ as a key initiator in the synaptic dysfunction. Instead, the link between Aβ and tau is evident as the oligomeric Aβ causes the mislocalization of tau, leading to synaptic dysfunction [31,32]. This view is reinforced by findings in a mouse model with β-amyloid plaque deposition, in which behavioral impairments and excitotoxicity associated with Aβ are reduced owing to a tau null background [33]. Recent findings by Ittner et al., also shed light to the possibility that hyperphosphorylated tau postsynaptically mediates the Aβ-induced toxicity, further emphasizing the reciprocal nature of the tau- and Aβ-mediated deleterious effects on synapses [32].

### Aβ-induced LTD activation via regulation of AMPAR localization

**Figure 1:**
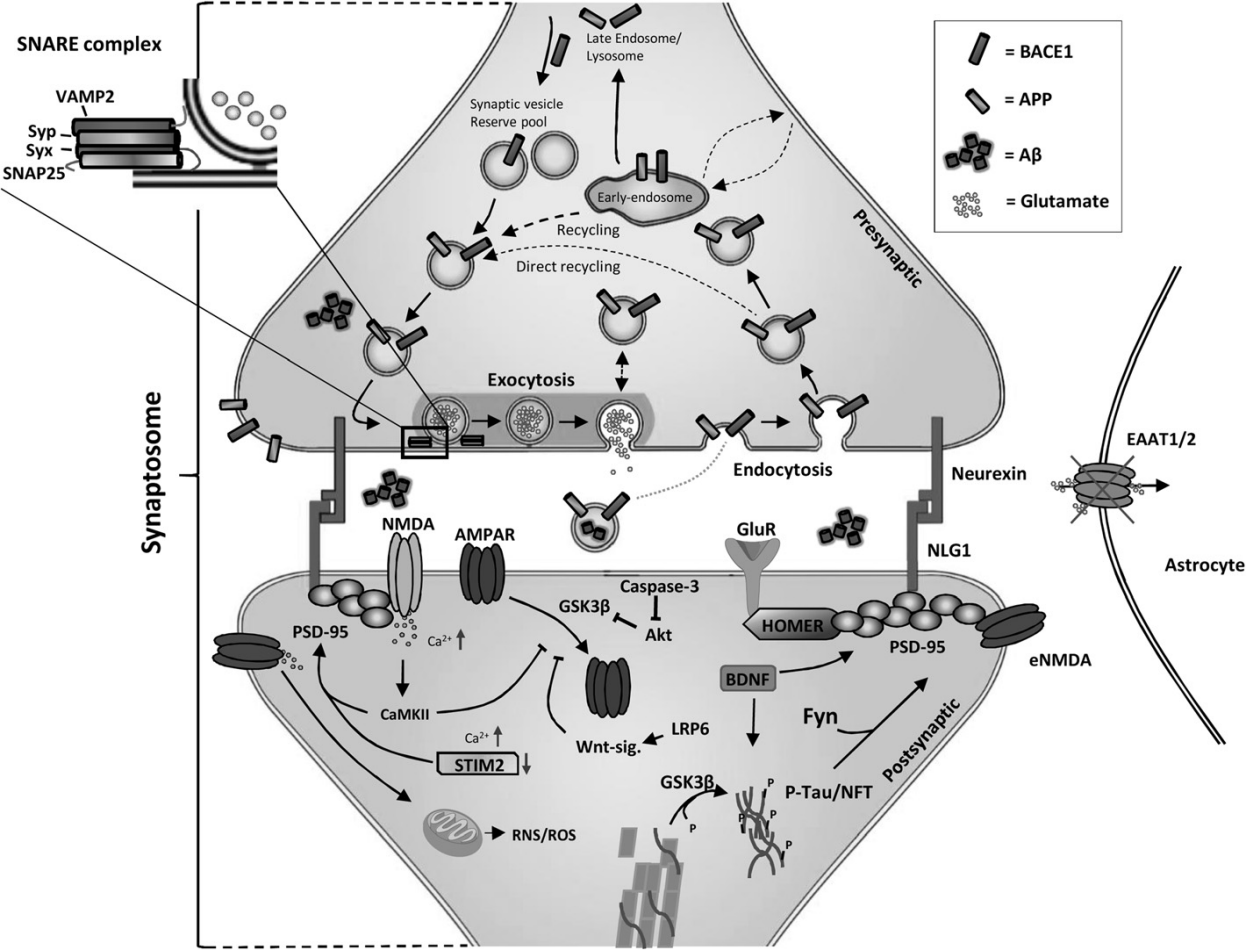
**Schematic representation of specific synaptic alterations induced by excess accumulation of soluble Aβ.** Aβ is produced from APP through sequential cleavages by BACE1 and γ-secretase at the presynaptic site and released to the synaptic cleft. Increased Aβ accumulation results in the internalization of AMPAR from the postsynaptic membrane, possibly via caspase-3-Akt1-GSK3β or altered LRP6-mediated Wnt signaling. Aβ may induce activation of extrasynaptic NMDAR (eNMDAR), due to faulty EAAT1/2-mediated regulation of glutamate levels by astrocytes, leading to the activation induction of downstream RNS/ROS-mediated neurodegenerative events. Additionally, Aβ accumulation induces tau localization to postsynaptic sites, resulting in the postsynaptic recruitment of Src kinase Fyn. Aβ is also proposed to activate histone deacetylase 2, resulting in the suppressed expression of genes required for synaptic function and stability, such as *BDNF, Cdk5, Homer1, NLGN1, Syp, GluR1, GluR2, NR2A, NR2B,* and *STIM2*. Abbreviations: Brain-derived neurotrophic factor (BDNF), Cyclin-dependent kinase 5 (CDK5), Homer homolog 1 (Homer1), Neuroligin 1 (NLGN1), Synaptophysin (Syp), Glutamate receptor 1 (GluR1), Glutamate receptor 2 (GluR2), N-mehtyl-D-Aspartate 2A (NR2A), N-mehtyl-D-Aspartate 2B (NR2B), Stromal interaction molecule 2 (STIM2).

The processing of APP through the amyloidogenic pathway is increased in AD, and toxic forms of Aβ accumulate in the brain. Also, especially in the sporadic, late onset AD patients, decreased Aβ clearance is centrally associated with the accumulation of Aβ [34]. Concomitant to the increase in the toxic forms of Aβ, disturbances in signaling pathways mediated by caspase-3, Wnt, and GSK3β have been reported [35-38] (Figure 1). Many of these pathways suggest that AD may represent a form of metabolic disease in the brain with resistance or deficiency of brain insulin and insulin-like growth factor-1 [39-41]. GSK3β is a well-known player in AD, strongly associated with the formation of NFTs via hyperphosphorylation of tau. Recently, GSK3β has also been linked to AMPAR trafficking and synaptic plasticity and it is a necessary component for LTD induction [38] (Figure 1). During the induction of LTD, GSK3β is activated by protein phosphatase 1 via dephosphorylation of GSK3β Ser9 [38]. Active GSK3β is known to co-localize with AMPAR, implying that it regulates the trafficking of AMPAR from the postsynaptic membrane [38]. On the other hand, caspase-3 may cleave Akt1, rendering Akt1 incapable of inhibiting GSK3β activation [36]. In the presence of Aβ, caspase-3 is activated and it cleaves Akt1. This allows the activation of GSK3β by PP1, and possibly the subsequent removal of AMPARs from the postsynaptic membrane, resulting in LTD induction [36] (Figure 1).

Aβ-induced synaptic dysfunction can also be mediated via impairment of the Wnt signaling pathway (Figure 1). Wnt signaling takes part in the modulation of several neuronal processes, such as neurotransmitter release at the presynaptic terminal, glutamate receptor trafficking and interactions with postsynaptic density protein 95 (PSD-95), which are the key components in LTP and LTD [42,43]. Recently Liu et al., 2014 [29] focused on the relationship between Wnt signaling and AD. They found that low-density lipoprotein receptor-related protein 6 (LRP6)-mediated Wnt signaling is downregulated in post-mortem AD brains and that it negatively correlates with Aβ levels. Reduced LRP6-mediated Wnt signaling may not only lead to synaptic dysfunction, but also to an increase in the amyloidogenic processing of APP, creating a vicious cycle between increased production of Aβ and decreased LRP6-mediated Wnt signaling in AD pathogenesis [37]. The exact molecular mechanisms related to synaptic dysfunction due to the decrease in LRP6-mediated Wnt signaling is not known, but it can be hypothesized that LRP6 could be a relevant factor for the maintenance of glutamate receptors at the postsynaptic membrane and thus induction of LTP [37,42,43].

### Aβ-induced overstimulation of extrasynaptic NMDAR

It has been suggested that LTP disruption in AD could also be mediated through Aβ-induced overstimulation of extrasynaptic NMDAR, due to impaired regulation of glutamate levels (Figure 1). This may lead to calcium dyshomeostasis and different redox events [7,11,23,44]. In AD, glutamate transporters EAAT1 and EAAT2, which are responsible for glutamate uptake in glial cells, are downregulated in the brain of AD patients [43]. This results in excess accumulation of glutamate to the synaptic cleft, and overstimulation of NMDAR. Increased Aβ levels have also been shown to cause astrocyte-mediated glutamate release, which can further exacerbate the excitotoxicity [11] (Figure 1). Ultimately, overstimulation of NMDAR triggers various translational and post-translational modifications in a vast set of proteins, resulting in the activation of downstream pathological events [7,45]. Supporting the relevance of NMDAR overstimulation in AD, a partial NMDAR antagonist, which blocks the NMDA overstimulation, has been shown to be neuroprotective in various animal models and to alleviate both neurodegenerative and vascular processes [46-48]. Memantine, a partial NMDAR blocker is also used as a treatment for AD patients and it has a beneficial impact in Parkinson’s disease (PD) patients. Unfortunately, memantine only provides short-term relief, indicating that there are several underlying mechanisms contributing to synaptic dysfunction in these disorders [49-51].

### Mislocated tau mediates AD-related synaptic deficiency

Tau has recently been identified as a mediator of Aβ-related excitotoxicity [32]. Tau is a microtubule-stabilizing axonal protein, but it is also known to function in the dendritic compartments with a pivotal role in postsynaptic plasticity [31,32,52]. At resting-state, tau is widely spread throughout the dendrites from where it is transported to postsynaptic sites upon synaptic activation. Activity-dependent tau translocation simultaneously induces an increase in LTP-related molecular components, such as PSD-95, glutamate receptor subunit GluR1, and Fyn, at the postsynaptic site [31]. Moreover, augmented Aβ levels have been shown to increase the localization of tau to postsynaptic sites during the resting-state and to disrupt the recruitment of PSD-95 and GluR1 during synaptic activation [31]. These results collectively suggest that tau is an important functional constituent sustaining LTP. This concept is in line with the findings showing Aβ-related reduction in LTP activation [53]. The abnormal localization of tau has been especially observed in mice expressing the full-length P301L mutant of tau [32,54]. Due to the aberrant resting-state localization, tau is likely able to enhance Aβ-related excitotoxicity by promoting the localization of the Src kinase Fyn to the postsynaptic sites [32]. Fyn is responsible for the phosphorylation of NMDAR subunit 2B (NR2B), which again facilitates the interaction between NR2B and PSD-95 [55,56]. Disruption of the NR2B/PSD-95 complex has been shown to prevent the excitotoxic effects of Aβ, suggesting that tau-dependent Fyn localization to the postsynaptic site plays a key role in Aβ-related synaptic dysfunction [32,57]. Furthermore, the fact that APP23/*tau*^*-/-*^ mice show significantly reduced premature mortality and susceptibility to Aβ-related excitotoxicity as compared to APP23 mice provides further support for the idea that tau mediates Aβ-related excitoxicity. Conversely, APP23 mice expressing the full-length P301L mutant tau show increased premature mortality [32]. Collectively, these findings highlight tau as a plausible target for intervention in AD apart from Aβ.

### Epigenetic changes in synaptic plasticity-related genes in Alzheimer’s disease

Other possible pathogenic mechanisms by which Aβ accumulation may cause synaptic dysfunction in AD are epigenetic alterations. Recent findings show that Aβ induces epigenetic changes via an increase in histone deacetylase 2 (HDAC2) levels, leading to decreased expression of *Arc*, *BDNF*, *Cdk5*, *Erg1*, *Homer1*, *NLGN1*, *Syp*, *GluR1*, *GluR2*, *Nfl*, *NR2A*, *NR2B*, *STIM2*, and *Syt1* [58]. These are essential presynaptic and postsynaptic components for synaptic plasticity (Figure 1) [6,59-62]. It has been observed that RNA interference (RNAi)-mediated reduction of HDAC2 levels in p25 overexpressing mice results in rescued synaptic morphology and plasticity. The reduction of HDAC2 in p25 overexpressing mice also results in the alleviation of cognitive and memory functions [58]. To further prove the relevance of HDAC2 in AD, post-mortem samples of AD brain were analyzed to show that HDAC2 accumulation was evident already at early stages of the disease progression [58]. This further underscores the notion that there are several underlying mechanisms contributing to synaptic dysfunction in neurodegenerative diseases.

The above-mentioned factors and pathways are only some of which may mediate Aβ-induced synaptic dysfunction. Several other factors, which are essential for synaptic function and possibly affected by Aβ, such as PSD-95, α7nAChR, PrP^c^, have also been identified. This indicates that Aβ may induce synaptic dysfunction in AD via a very complex combination of different mechanisms [63-65]. Further studies are needed for a complete understanding of the complex array of different pathways regulating synaptic function in health and disease.

### Altered synaptic plasticity in Parkinson’s disease

AD-related synaptic dysfunction has been widely studied as it is clearly connected to neurodegeneration and brain atrophy in AD patients. However, dysfunction and degeneration of synapses is a common hallmark of also other neurodegenerative disorders, such as Parkinson’s disease (PD), Huntington’s disease (HD), and frontotemporal lobar degeneration (FTLD) [66-69]. PD is a progressive, debilitating neurodegenerative disorder characterized by deterioration of motor capacities, and in some cases, dementia [70]. The main clinical hallmarks of PD are progressive loss of *substantia nigra pars compacta* neurons and formation of Lewy bodies/neurites in the substantia nigra, the brain stem and the cerebral cortex. A subset of PD cases are caused by mutations in genes, such as *α-synuclein*, *parkin*, and *LRRK2* [71-73]. A decrease in LTP activation has been shown in PD models. There is also evidence that treatment with a dopamine precursor alleviates the reduced LTP [3,68]. The underlying cause for the reduced LTP remains elusive, but a relationship between the above mentioned genetic determinants and presynaptic function has been suggested. Mutated α-synuclein, a main component of Lewy bodies found in post-mortem PD brain, is known to localize to presynaptic terminals and may negatively impact synaptic vesicle (SV) docking and release [10,74,75]. Burre et al., 2010 [61] showed that α-synuclein interacts with vesicle-associated membrane protein 2 (VAMP2), a N-ethylmaleimide-sensitive fusion protein receptor (SNARE) complex protein, and stabilizes the SNARE complex during synaptic activity. This agrees with the findings that mutations in α-synuclein lead to toxic effects and result in reduced neurotransmitter release in hippocampal pyramidal neurons [10]. Furthermore, overexpression of mutated α-synuclein decreases the levels of synapsin and complexin 2, corroborating the idea that α-synuclein mutations lead to impaired SV release [10]. Also, co-immunoprecipitation of Rab 3A and the A30P α-synuclein mutant implicate a link between SVs and α-synuclein [76,77]. LRRK2, another important player in PD, has also been hypothesized to regulate SV recycling, but through endocytic processes [78-80]. LRRK2 is suggested to colocalize with Rab5b and thus alter endocytic vesicular transport, suggesting that LRRK2 may affect SV trafficking [79]. Several findings support the notion that LRRK2 alters endocytosis and overexpression of LRRK2 has been shown to suppress SV endocytosis in mouse primary hippocampal neurons [80]. These data altogether suggest that deficient synaptic function plays an important role in the pathogenesis of PD.

### Alterations in synaptic activity in Huntington’s disease and frontotemporal lobar degeneration

Altered synaptic plasticity may also be involved in the pathogenesis of HD, a neurodegenerative disorder causing motor dysfunction, psychiatric symptoms, and cognitive decline [81,82]. Evidence shows that impairment of LTP is evident in HD [2,69]. It has been suggested that increased glutamate release associated with this disease results in the overexcitement of post-synaptic glutamate receptors [69]. HD is caused by the expansion of a CAG repeat in the *Htt* gene, which leads to the aggregation of Htt protein to the nucleus and cytoplasm of cells. This has toxic effects and eventually leads to cell death [82]. The mechanisms by which the *Htt* mutation causes neurodegeneration remain so far elusive. However, altered neuronal activity can be initially noticed in the cortex and striatum of HD brain, from where it further spreads to other brain areas and leads to neuronal degeneration [82]. As mentioned above, an increase in glutamate release is apparent at the early stages of HD, and this eventually results in the loss of glutamatergic terminals. A reduction in astrocyte glutamate transporter 1 (GLT1) levels is commonly observed in HD rodent models, which could contribute to the accumulation of excess glutamate [83-85]. Excess glutamate will most likely lead to stimulation of extrasynaptic NMDAR, leading to the activation of downstream neurodegenerative events [86]. Supporting this notion, extrasynaptic NMDAR expression and signaling are increased in acute brain slices and corticostriatal cultures from the HD mouse model YAC128. Moreover, clinical studies have indicated that the partial NMDAR blocker memantine has beneficial effects in HD patients [87].

Studies of synaptic alterations in FTLD have been so far limited. FTLD is heterogeneous group of clinical syndromes, which leads to dementia and primarily affects the frontal and temporal lobes of the brain [81]. Evidence for synapse loss and a decrease in synaptic density in FTLD brains implicates that synaptic dysfunction may also underlie the pathogenesis of this disease entity [88-90], but no clear explanation for these synaptic alterations have been found. Alterations in synaptic proteins are also evident at specific layers of the frontal cortex in FTLD brain, pointing towards the possibility that a decrease in synaptic activity could underlie the clinical outcome [67]. In conclusion, eminent data implicate that altered synaptic function is centrally involved in the early pathogenesis of the different neurodegenerative diseases. Although diverse brain regions are specifically affected in each of these diseases, the current data suggest that common molecular mechanisms leading to synaptic dysfunction may underlie disease pathogenesis. Therefore, characterization of factors and pathways, which regulate synaptic function, is essential and may lead to the discovery of novel therapeutic targets.

### The Septin protein family

Alterations in the functions of neuronal cells is evident in neurodegenerative diseases. Especially, changes in the synaptic plasticity during early phases of these diseases have been suggested to lead to the activation of neurodegenerative events. The complexity of the mechanisms of synaptic plasticity complicates the understanding how these processes are altered in different disorders. However, understanding the mechanisms leading to deficient function and degeneration of synapses is essential for a better comprehension of the pathogenesis of neurodegenerative diseases in general. One of the potential candidates regulating synaptic function is the septin protein family.

**Figure 2:**
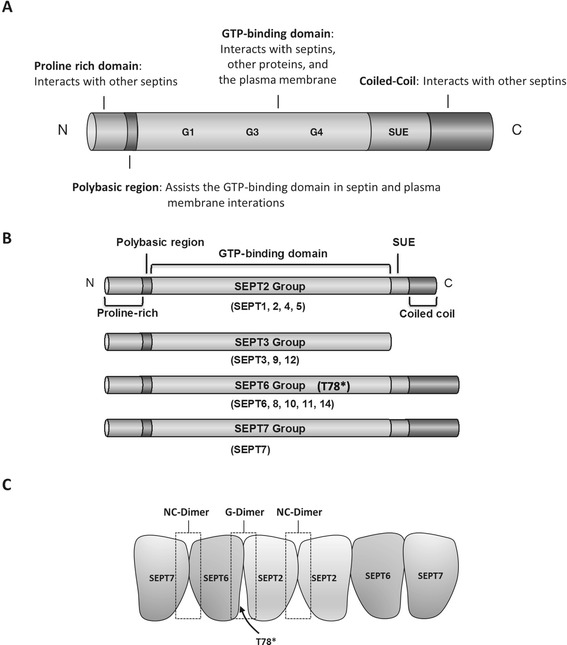
**Schematic showing the common structure of septin protein family and the structures of individual septin subgroups. A**. The septin protein structure consists of a GTP-binding domain composed of conserved motifs G1 (GxxxxGK[S/T]), G3 (DxxG) and the GTP-specificity motif G4 (xKxD). D, G, K, S, and T represent aspartic acid, glycine, lysine, serine, and threonine residues, respectively, and x indicates any amino acid. The N-terminus consists of a proline-rich domain and a polybasic region. The C-terminus contains a septin unique element (SUE) and a varying α-helical coiled-coil domain. **B**. Based on sequence homology and domain composition, the 13 septins have been divided in to four subgroups (SEPT2, SEPT3, SEPT6, and SEPT7). Septins of subgroup SEPT6 lack a threonine residue (T78*), which is needed for the hydrolyzation of GTP to GDP. **C**. The formation of septin filaments mediated by the interaction between GTP-binding domains (G-dimer) and the N- and C-termini containing faces (NC-dimer). Formation of septin filament structures require different conformational changes mediated by the GTP/GDP molecules, allowing the assembly and disassembly of stable septin complexes. These conformational changes are also influencing the N-terminal helix and thus affect the formation of the NC-dimer. Therefore, the lack of a threonine residue (T78*), resulting in the inability of the septin protein to hydrolyze GTP to GDP, enables the formation of e.g. SEPT2-6-7 complex.

Septins are a highly conserved family of GTP-binding proteins [91,92]. In mammals, there are 13 known septins, which are divided in to four subgroups; SEPT2 (Septin 1, 2, 4, 5), SEPT3 (Septin 3, 9, 12), SEPT6 (Septin 6, 8, 10, 11, 14), and SEPT7 (Septin 7) [12]. The septin protein family members are highly expressed in the brain, and are known to take part in processes such as regulation of formation, growth and stability of axons and dendrites, synaptic plasticity, and vesicular trafficking [12,92-96]. In addition to these physiological functions, septins have been linked to different neurodegenerative and psychiatric disorders, such as PD, AD, and schizophrenia [97-99]. The septins are 30-65-kDa proteins, and they share a common central GTP-binding domain, 53 highly conserved amino acids known as the septin unique element (SUE) at the C-terminus, and a polybasic region located at the immediate N-terminus (Figure 2). The GTP-binding domain consists of the conserved α-β core, built up by interacting α-helices and β-strands, and the loop elements, which take part in the binding of GTP and its possible hydrolysis to guanosine diphosphate (GDP). The GTP-binding domain also contains two α-helical elements at the ends of the conserved core and two insertions, one α-helix and one β-hairpin, in the GTPase core [100-102]. This domain mediates the formation of septin filaments and interactions with various other proteins. The neighboring polybasic region is believed to assist the GTP-binding domain in associations with other septins and is capable of directly binding to the phosphoinositides on the plasma membrane. The functions of the neighboring SUE are so far unknown. The rest of the protein is composed of varying N- and C-terminal regions, which may contain a proline-rich domain and an α-helical coiled-coil domain, respectively. Many of the septins possess α-helical extension at their N- and C-termini. The N- and C-terminal regions also play a vital role in septin interactions [91,103,104].

The role of GTP and GDP in the function and assembly of septin filaments is still fairly poorly known. Evidence shows that the presence of GTP regulates the positions of structural motifs in the GTP-binding domain called switches. The different conformational changes caused by the GTP/GDP molecules are believed to be required for the formation of stable septin complexes and the dissociation of the complexes during different phases of the cell cycle [105]. These conformational changes are also transmitted through to the N-terminal helix affecting the septin-septin interactions. Therefore septins subgroup SEPT6 (SEPT6, 8, 10, 11, and 14) lack a threonine residue (T78*), which is needed for the hydrolyzation of GTP to GDP [105]. This feature is necessary for the formation of certain complexes, such as the SEPT2-6-7 trimeric filament, where GTP can stabilize the SEPT2-6 GTP-binding domain interaction (G-dimer), without affecting the SEPT6-7 N- and C-termini interaction (NC-dimer) [100] (Figure 2). A further role in the formation, localization and function of septin complexes has been hypothesized for GTP and GDP. No direct evidence for this has been found, but mutations in residues at the GTP-binding site have been shown to alter these features [101].

### Septins in neurodegenerative disease-related synaptic processes

The septin family provides several interesting candidates possibly involved in the underlying mechanisms of synaptic dysfunction and neurodegeneration in neurodegenerative diseases. Septins have been shown to associate with AD, PD, HD, FTLD, and Down syndrome [98,99,106-111], suggesting that septins are involved in the pathogenic mechanisms of different neurodegenerative diseases. Related to this, a recent study on the brain proteome revealed that SEPT2/3 levels were increased, while SEPT5 levels were decreased in the temporal neocortex of AD patients as compared to non-AD subjects [112]. Also, genetic characterization of *SEPT3* gene identified a polymorphic site at the exon 11, which significantly associated with AD in a case-control study [113]. Moreover, studies in the frontal cortex homogenates of FTLD-U patients have shown an increase in the truncated forms of SEPT11 (~45 kDa, ~37 kDa, and ~28 kDa) and the presence of fibrillar thread-like structures of SEPT11, which were specifically localized to the superficial cortical layers [108]. The pathological functions of these thread-like structures remain elusive, but based on the known localization of SEPT11 to microtubules and stress fibers, it can be hypothesized that formation of these structures could disrupt cytoskeletal functions and result in cellular toxicity [114,115]. In the context of HD, a significant down-regulation of SEPT4 was detected in cells expressing mutated huntingtin [116].

**Figure 3:**
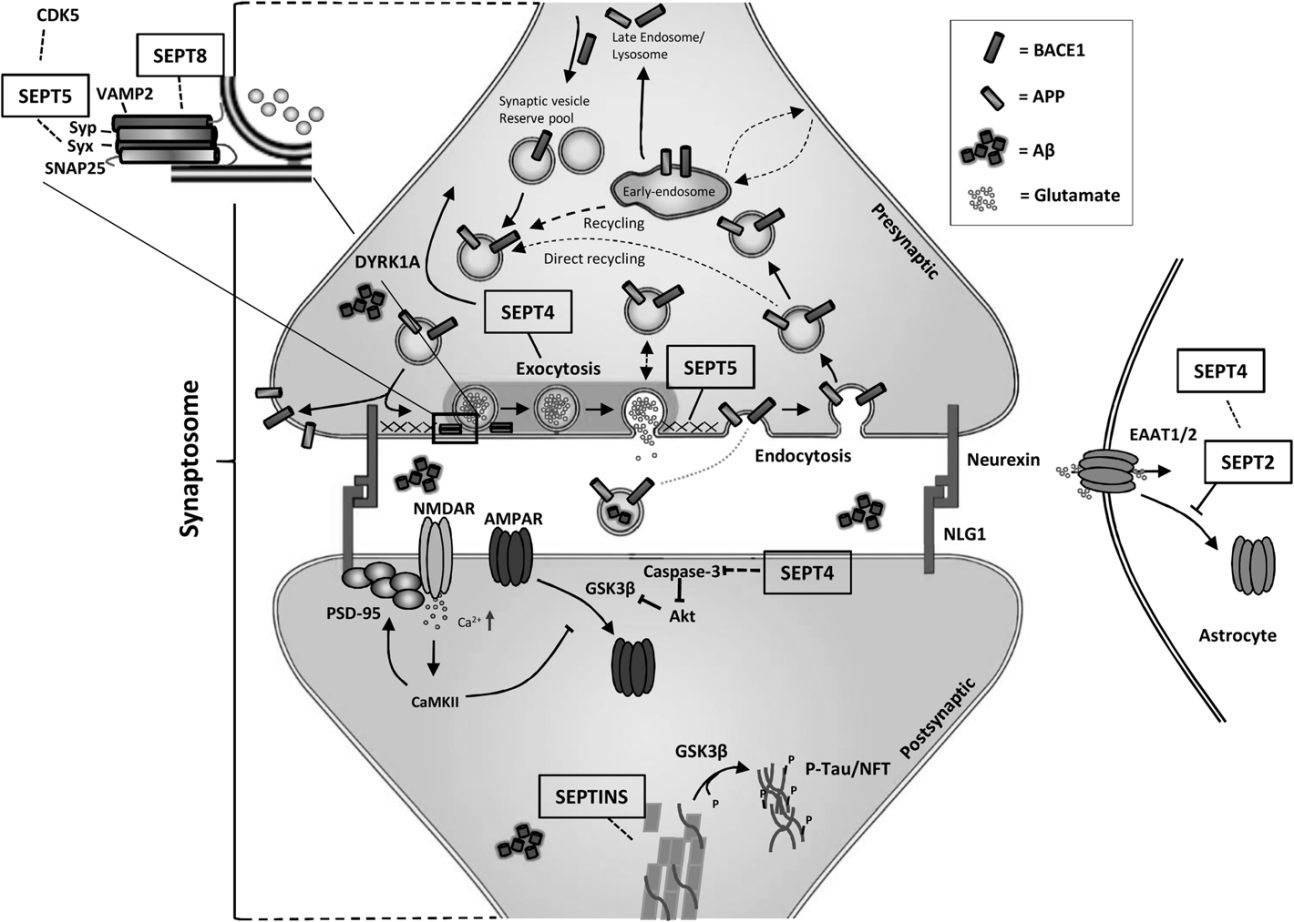
**The possible roles of septins in synaptic plasticity and mechanisms of neurodegeneration.** SEPT4, SEPT5 and SEPT8 have been hypothesized to control synaptic vesicle trafficking at the presynaptic terminal by interacting with different components of the SNARE complex and regulating synaptic vesicle localization at the presynaptic membrane. Also SEPT2 and SEPT4 may regulate neurotransmitter levels at the synapse by regulating glutamate transporter localization in astrocytes. Additionally, SEPT4 may affect caspase-3 activity. SEPT4 functions are possibly regulated by DYRK1A-mediated phosphorylation. Septins also are known to interact with actin and microtubules, suggesting that they may affect tau. SEPT1, 2, and 4 have been found to localize in in NFTs.

The diverse interactome of septins suggests that they have an essential role in vesicle trafficking, which may be key for e.g. sufficient neurotransmitter release. SEPT8 has been shown to interact with components, such as VAMP2 and syntaxin-1 of the SNARE complex. SEPT8 possibly promotes the release of VAMP2 from synaptophysin during action potential stimulation, allowing the formation of the SNARE complex and subsequently enhanced docking of vesicles to the presynaptic membrane [94] (Figure 3). Conversely, SEPT5 has been suggested to negatively regulate SV release at inhibitory presynaptic terminals by forming filamentous barricades at the presynaptic membrane [92]. SEPT5 has also been shown to interact with syntaxin-1, resulting in decreased exocytosis [117,118] (Figure 3). SEPT5-syntaxin-1 interaction and the formation of filamentous barricades is considered to be regulated by the CDK5-mediated changes in SEPT5 phosphorylation status [93]. CDK5 is able to phosphorylate SEPT5 at serine 161 (S161) and 327 (S327). Blocking the phosphorylation of SEPT5 at these sites resulted in enhanced binding of SEPT5 to syntaxin-1 in PC12 cells [93]. The activity of CDK5 is deregulated in AD [119]. This could result in altered SEPT5 phosphorylation and exocytosis at inhibitory presynaptic terminals, and thus possibly contribute to altered synaptic activity in AD. Parkin 2 (PARK2), an E3-ubiquitin ligase, has been identified as another possible modulator of SEPT5-syntaxin-1 interaction. Interestingly, mutations in *PARK2* are responsible for autosomal recessive early-onset PD and a subset of sporadic PD [71]. PARK2 ubiquitinates SEPT5, which leads to the degradation of SEPT5, enabling the release of syntaxin-1 to enhance SV docking [111]. This agrees with the idea that PD-associated reduction in parkin results in the accumulation of SEPT5 and subsequent neuronal toxicity in dopaminergic neurons [107,120].

The fact that SEPT1, 2, and 4 have been found in NFTs provides further confirmation that different septin family members are associated with neurological diseases [98,110]. SEPT2 is involved in synaptic plasticity and has been found to interact with the glutamate transporter EAAT1 and regulate its cellular localization (Figure 3). SEPT2 binding to EAAT1 is GDP-dependent and GDP-bound SEPT2 is capable of binding to and internalizing EAAT1. GDP binding to septins is considered to disrupt the formation of septin filaments and thus it is hypothesized that the GTP-bound form of SEPT2 is capable of forming septin filaments and stabilizing EAAT1 at the cell surface [100,121]. The absence of EAAT1 from the cell surface could lead to the increased levels of glutamate in the extracellular space and possibly overstimulation of NMDAR [121] (Figure 3). Co-immunoprecipitation studies of SEPT2 and EAAT1 have revealed that also SEPT4 interacts with EAAT1, which points to a possible formation of heteromeric complexes between SEPT2 and SEPT4 [121]. In addition to the co-localization with NFTs and EAAT1, SEPT4 has been linked to PD and Down syndrome through interaction with parkin and DYRK1A (dual-specificity tyrosine phosphorylation-regulated kinase 1A) [106,122]. DYRK1A levels are known to be increased in Down syndrome patients and it has been shown to phosphorylate SEPT4 at S68 and S107. The direct impact of this phosphorylation is elusive, but DYRK1A also phosphorylates α-synuclein, which is another interacting partner of SEPT4 [122]. Since α-synuclein is the key component of PD-related Lewy bodies, the DYRK1A-mediated phosphorylation of SEPT4 may be associated with the formation of Lewy bodies [109,122]. Loss of SEPT4 in dopaminergic neurons has been observed in the sporadic PD patients, which could be due to the sequestration of SEPT4 into α-synuclein aggregates and neuronal loss [109,123]. The loss of SEPT4 also results in diminished dopaminergic neurotransmission, suggesting that SEPT4 may play a central role in dopamine release and reuptake in the presynaptic machinery [109]. SEPT4, similarly to SEPT5, is also a substrate for parkin, emphasizing further the potential importance of SEPT4 in the pathogenesis of PD [106]. Furthermore, SEPT4 may be involved in AD based on its interactions with X-linked inhibitor of apoptosis protein, a regulator of caspase-3 activity [124,125] (Figure 3).

Overall, considering the localization of septins in neurons, their involvement in the regulation of synaptic functions, and their other known interactions, septins may prove as central candidates involved in the pathogenic mechanisms of various neurological diseases. However, further studies are need to comprehensively understand septin functions and the outcomes of septin interactions. A focus should also be set on understanding the effects of septin-septin interactions, because they are known to form various hetero- and homomeric structures, which have regulatory and compensatory effects on neuronal functions [104]. Recent findings by Tokhtaeva et al. further emphasize the importance of studying septin-septin interactions, since disabling the formation of heteromeric septin oligomers impairs the exocytosis of proteins and neurotransmitters [126]. It was also shown that septins undergo constant reassembly at different phases of vesicle recycling, supporting their role in the various steps related to neurotransmitter release and uptake [126].

## Conclusion

The underlying mechanisms in different neurodegenerative disorders have remained elusive. However, increasing evidence suggests that abnormal synaptic activity and synaptic dysfunction are common in different neurodegenerative diseases and may in fact represent some of the earliest pathogenic alterations during their pathogenesis. In agreement with this notion, recent studies have shown that changes in the expression levels of specific synaptic proteins in the cerebrospinal fluid reflect degeneration of synapses and can be successfully used to predict AD patients and evaluate MCI-to-AD conversion at very early stages of the disease [127,128]. The observation that the protein levels of different septins are altered in AD patients [114] suggests that also septins could represent early markers linked to synaptic dysfunction and synaptoxicity. Alterations in the expression levels, phosphorylation status, and subcellular localization of various pre- and postsynaptic proteins in neurodegenerative diseases emphasize that extremely complex mechanisms are likely to be involved in the etiology of these diseases. Therefore, further research is needed to unravel the specific mechanisms by which synaptic plasticity is affected in neurodegenerative diseases. Thus, it is plausible that novel disease biomarkers and therapeutic targets will be identified through more detailed characterization of the aberrant changes in synaptic plasticity-related factors and pathways at different phases during the progression of these diseases [129]. This may require identification of new candidates, which are involved in the regulation of synaptic plasticity and neurodegenerative disease-related mechanisms. Septin protein family, implicated in the regulation of several different aspects of synaptic vesicle trafficking and neurotransmitter release, may offer such novel candidates for further assessments in the pathogenesis of neurodegenerative diseases. Therefore, the future studies should focus on better understanding of the functions, regulation, and interactomes of different septin family members in health and disease.

## Abbreviations

AD: Alzheimer’s disease
AMPAR: AMPA receptor
APP: Amyloid precursor protein
Aβ: Amyloid-β
BDNF: Brain-derived neurotrophic factor
CDK5: Cyclin-dependent kinase 5
DYRK1A: Dual-specificity tyrosine phosphorylation-regulated kinase 1A
FTLD: Frontotemporal lobar degeneration
GluR1: Glutamate receptor 1
GluR2: Glutamate receptor 2
GLT1: Glutamate transporter 1
GDP: Guanosine diphosphate
GTP: Guanosine triphosphate
HDAC2: Histone deacetylase 2
Homer1: Homer homolog 1
HD: Huntington’s disease
LTD: Long term depression
LTP: Long-term potentiation
LRP6: Low-density lipoprotein receptor-related protein 6
SNARE: N-ethylmaleimide sensitive fusion protein receptor
NR2A: N-mehtyl-D-Aspartate 2A
NR2B: N-mehtyl-D-Aspartate 2B
STIM2: Stromal interaction molecule 2
Syp: Synaptophysin
NFT: Neurofibrillary tangles
NLGN1: Neuroligin 1
NMDAR: NMDA receptor
PARK2: Parkin 2
PD: Parkinson’s disease
RNAi: RNA interference
SUE: Septin unique element
SV: Synaptic vesicle
VAMP2: Vesicle-associated membrane protein 2
